# Association between Protective and Deleterious *HLA* Alleles with Multiple Sclerosis in Central East Sardinia

**DOI:** 10.1371/journal.pone.0006526

**Published:** 2009-08-05

**Authors:** Roberta Pastorino, Cristina Menni, Monserrata Barca, Luisa Foco, Valeria Saddi, Giovanna Gazzaniga, Raffaela Ferrai, Luca Mascaretti, Frank Dudbridge, Carlo Berzuini, Salvatore Bruno Murgia, Maria Luisa Piras, Anna Ticca, Pier Paolo Bitti, Luisa Bernardinelli

**Affiliations:** 1 Dipartimento di Scienze Sanitarie Applicate e Psicocomportamentali, Università di Pavia, Pavia, Italy; 2 Dipartimento di Statistica, Università di Milano Bicocca, Milano, Italy; 3 Centro di Tipizzazione Tissutale, S.I.T., Presidio Ospedaliero S. Francesco, ASL N°3, Nuoro, Italy; 4 Divisione di Neurologia, Presidio Ospedaliero S. Francesco, ASL N°3, Nuoro, Italy; 5 Blood Transfusion Centre, San Gerardo Hospital, Monza, Italy; 6 MRC Biostatistics Unit, Institute of Public Health, University Forvie Site, Cambridge, United Kingdom; 7 Department of Pure Mathematics and Mathematical Statistics, University of Cambridge, Cambridge, United Kingdom; Tokyo Medical and Dental University, Japan

## Abstract

The human leukocyte antigen (HLA) complex on chromosome 6p21 has been unambiguously associated with multiple sclerosis (MS). The complex features of the HLA region, especially its high genic content, extreme polymorphism, and extensive linkage disequilibrium, has prevented to resolve the nature of HLA association in MS. We performed a family based association study on the isolated population of the Nuoro province (Sardinia) to clarify the role of HLA genes in MS. The main stage of our study involved an analysis of the ancestral haplotypes A2Cw7B58DR2DQ1 and A30Cw5B18DR3DQ2. On the basis of a multiplicative model, the effect of the first haplotype is protective with an odds ratio (OR) = 0.27 (95% confidence interval CI 0.13–0.57), while that of the second is deleterious, OR 1.78 (95% CI 1.26–2.50). We found both class I (A, Cw, B) and class II (DR, DQ) loci to have an effect on MS susceptibility, but we saw that they act independently from each other. We also performed an exploratory analysis on a set of 796 SNPs in the same HLA region. Our study supports the claim that Class I and Class II loci act independently on MS susceptibility and this has a biological explanation. Also, the analysis of SNPs suggests that there are other HLA genes involved in MS, but replication is needed. This opens up new perspective on the study of MS.

## Introduction

Multiple sclerosis (MS) is an autoimmune disease which mostly affects young people and which causes myelin destruction and neuronal cells degeneration leading the patient to a progressive disability. The disease has a preference for females and may manifest relapsing or progressive forms. Although the picture of genes involved in the susceptibility to MS is far to be completed, the Human Leukocyte Antigen (*HLA*) complex on chromosome 6p21 has been unambiguously associated with MS [1]–[8].

*HLA* Class I and Class II genes code proteins which are central in activating antigen-specific immune responses. *HLA* Class I and Class II proteins bind antigenic peptides and display them on the cell surface for recognition by CD8+or CD4+T-lymphocytes, respectively, thus activating the cellular immune response [9]. Many of the *HLA* associated disease are believed to be autoimmune [10]. Indeed among mature lymphocytes, there are many potential autoreactive T and B cells [11]. In MS, improper activation of the immune system in response to myelin antigens is thought to be a major cause of inflammatory deyemelination. Initially the association between *HLA* and MS was studied in *HLA* Class I loci [12]. However, the attention turned to *HLA* Class II loci as Class I loci were presumed to be secondary to Linkage Disequilibrium (LD) with disease predisposing Class II alleles [13]–[15]. Consistent results were found particularly for *HLA*-*DRB1* and *HLA*-*DQB1*. Indeed, the *HLA*-*DR2* haplotype is one of the most reproduced findings in the genetics of the Major Histocompatibility Complex (MHC) [3], [5], [6], [13], [16]–[18]. The *HLA*-DRB1*1501 allele, encompassed within the *DR2* specificity, was found to be strongly associated with MS in North American, in North European Caucasian patients and in White Brazilians from Rio de Janeiro and from Sao Paolo [13], [19]–[23], while the DRB1*1503 allele has been associated with MS in Mulattos from Sao Paolo and in Afro-American patients [21], [24], but not in Afro-Brazilians from Rio de Janeiro [19], [25]. The DRB1*0301, and DRB1*0401 alleles were seen to be over-represented in Sardinians [26]. In Turkish patients and in those coming from the Canary Islands, an association was reported between the *HLA*-DRB1*04 allele group and the disease [27]. The *DRB1*-*17* allele has long been known to be associated with susceptibility in the Swedish population [28] and this was confirmed in Canadians [29]. A recent pooled analysis [30] of the past 30 years of research on the role of MHC in multiple sclerosis, highlights the preeminent role of the extended haplotype defined by *HLA*-DRB1*1501 in MS. Two other ancestral haplotypes containing *HLA*-*DR3* and *HLA*-*DR4* also appear to play a role in MS susceptibility, although the effect of these haplotypes on disease is more modest than that of the *HLA*-*DR2* haplotypes.

Recently, the possible importance of *HLA* Class I genes in MS susceptibility has been reinvestigated and genetic association has been found. In Swedish patients, *HLA-A3* was positively associated to MS, independently on *DRB1-15*, while a negative association was found for *HLA-A2* [31], [32]. The same association was reported in Tasmanians [33]. In Norwegian sporadic MS patients association was seen with *HLA*-*A*, *HLA-B* [34]. Indeed, the *HLA*-*A3* gene was found to increase the risk conferred by the *HLA*-DRB1*1501 haplotype. Mapping studies from the UK and the US identified a significant protective effect of *HLA*-*CW5* [17].

Clearly, these results indicated that one allele for any given *HLA* locus, tends to exhibit different behaviours and confer resistance or susceptibility in response to different environmental or genetic backgrounds; within the *DRB1* gene there appears to be evidence for allelic heterogeneity particularly in non European populations [35]. This may help to refine the conceptual model of MS pathogenesis and suggests the possibility that a complex *trans DRB1* allelic interactions may determine the balance between susceptibility and resistance [20].

Despite the numerous studies carries out so far, the complex features of *HLA* region, especially its high genic content, extreme polymorphism and extensive LD, has so far prevented the resolution of the nature of *HLA* association in MS.

Here we present the results of a study on the association of *HLA* and MS carried out in the Nuoro province of Sardinia.

The Mediterranean island of Sardinia, and in particular the Nuoro province, is well suited to identify *HLA* loci associated with MS. Indeed, it has an MS prevalence four/five times higher than the Italian mainland, registering 1 case per 500 inhabitants [36]. Isolation, genetic drift and perhaps selection have contributed to a genetic differentiation, and the *HLA* loci distribution is characterised by a very high number of rare haplotypes and by a small number of very frequent haplotypes [37]. Seven of them have a frequency higher than 0.85% [38] and thus are ancestral haplotypes. The two most frequent ancestral haplotypes are *A30Cw5B18DR3DQ2* (14.6%), which has the strongest LD observed in the world [39], and *A2Cw7B58DR2DQ1* (6.0%). Since ancestral haplotypes are highly conserved at least between *HLA-B* and *DR*, they can be used as markers for all polymorphisms within the entire MHC region and allow mapping studies of known and unknown genes without requiring assumptions as to the mechanisms involved [40]. More information on ancestral haplotypes is given in the supporting material S1.

The main aim of our work is to identify which of the above mentioned alleles of *HLA* ancestral haplotypes confer genetic susceptibility/protection to MS in the population of the Nuoro province, Central East Sardinia. A second aim is to detect variants associated to MS independently on *HLA* loci *A*, *B*, *Cw*, *DR* and *DQ* via an exploratory analysis on a set of 796 Single Nucleotide Polymorphisms (SNPs) located in the *HLA* region.

## Materials and Methods

### Study Design

We carried out a family based association study. The study sample consisted of 628 individuals belonging to 213 small groups, which we called “nuclei”. Each nucleus was ascertained around an MS case, or proband, extracted from the MS register running in the province of Nuoro since 1995. Diagnoses of MS were in accord with Poser's criteria for clinical definite MS [41]. All individuals gave informed consent to their inclusion in this study, which was approved by the local Ethics Committee. The nuclei were classified into 3 types: type-1 nuclei consisting of a proband and of his/her parents, plus occasionally the proband's siblings (n = 69); type-2 nuclei consisting of the proband, of his/her spouse and of their children (n = 28), and type-3 nuclei consisting of a proband and of a corresponding unrelated control, matched by village of origin (n = 116). SNPs genotyping was performed at the Centre National de Genotypage, Evry, France, while HLA typing was done at “Centro di Tipizzazione Tissutale”, Nuoro, Italy. Typing and quality control procedures are described in the supporting material S2.

### Statistical Analysis

#### Pseudocases and pseudocontrols

To deal with different types of trios, we adopted the same approach described in Bernardinelli *et al.* [42] which we outline below.

In a classical trio study, an ascertained proband is genotyped along with his/her parents to form a proband-parent trio (Type 1). Our approach extends this design by including two further ascertainment schemes before called “nuclei”. In the first, a proband is genotyped along his/her spouse and possibly their offspring (Type 2). In the second, an isolated proband is recruited into the study to act as an isolated case with a corresponding controls, matched by village of origin (Type 3).

We regard a family-based association study as a special case of matched case/control analysis, where each nucleus contributes pseudocases and pseudocontrols. In type 1 nuclei, we define the two pseudocases to be the two parental *HLA* haplotypes transmitted to the proband and the two pseudocontrols to be the remaining two parental *HLA* haplotypes. In type 2 nuclei, we define the pseudocases to be the two *HLA* haplotypes found in the proband and the pseudocontrols to be those found in the spouse. We can also define pseudocases and pseudocontrols at a genotype level, where the two transmitted haplotype form the pseudocase genotype and the two untransmitted haplotypes the pseudocontrol genotype. Likewise in type 2 and type 3 nuclei the genotype of the proband acts as pseudocase genotype and that of the spouse/population control as pseudocontrol genotype. We pool the three types of nuclei into a joint analysis which looks for a phased genotype of variants that are more (or less) frequent in the pseudocase than in pseudocontrols. So, we perform the analysis at a genotype (phased) level where the genotype is formed by two haplotypes.

#### Haplotype reconstruction and missing genotype data

Haplotypes reconstruction is done using a Bayesian Monte Carlo approach. Briefly, given genotyping data at multiple linked loci, we estimate the unobserved haplotypic phase and impute any missing calls by combining information about population haplotypic frequencies with information about the neighbouring loci. Using Phase [43], we obtain all possible haplotypic configurations, each with a weight assigned according to a coalescent underlying population model, but without considering the structure of the family. The weights are then updated via evaluating the Mendelian consistency between the different haplotype configurations of the parents and the genotype of the child. In the extreme situation, the method gives a weight equal to zero for the configurations which are not Mendelian consistent with the observed child genotype and a weight equal to 1 when there is only one possible configuration which is consistent with the child genotype. For type 2 trios, the presence of the child has the important role of providing information that allows us to reduce the number of possible configurations in the parents thus reducing the uncertainty in haplotype reconstruction and missing data imputation. For type 3 nuclei, however, weights are not updated as there are no children. This reweighting procedure is implemented in our Genetic Association Downstream Analysis (GADA) software that allows outputting all the configurations of the pseudocase and pseudocontrol genotype for each nucleus along with its posterior weight. We call the output the reconstruction table. Refer to our paper [42] for a formal description of the approach and an example of reconstruction table. The reconstruction table can be analyzed via unconditional weighted logistic regression as suggested by Cordell [44]. Our analysis does not maintain the matched design, and would be conservative under population stratification.

We extended this approach to allow for missing genotype data. When genotypes are missing, we exploit linkage disequilibrium in the local region to impute missing genotype calls using information from the neighbouring loci. Specifically, we include additional loci in the haplotype, whose contribution to disease risk is not of interest, but which provide information on missing genotypes of interest. The set of haplotypes consistent with a given unphased genotype is then expanded to include those with all possible completions of a missing genotype, while maintaining Mendelian consistency. This will attenuate the impact of biased missingness, for example when a SNP is preferentially missing heterozygotes among either cases or controls.

### Analyses

#### Regression based association test

In the regression each pseudocase genotype can be considered as an independently observed response/covariate pair, where the response is 0 for pseudocase and 1 for a pseudocontrol and the covariate represents the wild type homozygote or the heterozygote or the mutant homozygote genotype. The use of the weighting option in packages such as STATA and R allows each configuration of the pseudocase/pseudocontrol genotype for each nucleus to enter in the logistic regression with a relative importance fixed by the corresponding posterior weight. This allows us to take into account the uncertainty in phase reconstruction and the imputation of missing data.

We analysed the data by fitting regression models both to investigate departure from the multiplicative model and to identify the effect model best fitting the data, and to perform hypothesis driven tests to study the association between HLA alleles and SNPs variants with MS.

We started by modelling the relationship between MS and the HLA genetics markers to try to underpin the transmission inheritance model. We fitted the weighted regression model at a genotype level by assuming the following genetic models: (a) not assuming any model, (b) a dominant, (c) a recessive and (d) multiplicative.

For fitting the model (a), we fitted a regression model to the genotype considered as a categorical (3 levels) variable, for model (b) we created a binary variable by aggregating the heterozygote with the mutant homozygote, for model (c) we created a binary variable by aggregating the wild type homozygote with the heterozygote genotype, and for model (d) we included the covariate representing the genotype (three categories) as a continuous variable.

We used a Likelihood Ratio Test (LRT) to check the departure from a multiplicative model (we compared model (d) with model (a)). If the p-value for the LRT is statistically significant, then we conclude that there is a departure from the multiplicative model. In this case we fitted the regression model by assuming the dominant and the recessive model and selecting the best model on the basis of the Wald test measuring the goodness of fit.

For whichever purpose the model was fitted, we estimated a parameter (log Odds Ratio - OR) measuring the effect of a given genotype versus all the others on the risk of disease. We embedded in the regression model a permutation scheme (permuting the case/control label) that protects from the possibility that the weighting scheme used in the regression does not rigorously correspond to a likelihood of a specific model of the data. To compute p-values (which we call empirical p-values), we applied a permutation scheme to the Wald's statistics of the genotype specific odds ratio parameter, to the Wald test measuring the goodness of fit of the model and to the likelihood ratio tests used to check (a) the departure from a multiplicative model and (b) to perform a conditional independent test (see below).

#### Independent Effect and Sole Variant Tests

Finally, we performed two hypothesis driven related conditional tests (*independent effect* and *sole variant tests*) to try to identify which variant, or variants, is solely and independently associated with the disease [45].

When we perform a *sole variant test* we compare a model including both ‘everything else’ and the ‘particular variant’ with a model including the ‘particular variant’. If the p-value for the LRT is not statistically significant we conclude that the particular variant is the ‘sole variant’, i.e. it is the only variant that matters. When we perform an *independent test* we compare via LRT a model in which ‘everything else’ and the ‘particular variant’ are included with a model including ‘everything else’. If a p-value for the LRT is statistically significant, then we conclude that this particular variant is associated independently on everything else. In both cases, ‘everything else’ refers to the local haplotypic background as determined by the markers under study.

We also performed a *conditional independence test* between the significant associated variants by comparing the model including both variants and a model including just one of the variant. A statistically significant p-value indicates that a variant is not conditionally independent on the disease given the other variant. The test compares via a LRT a model in which a variant that is statistically significant and a model in which further variant(s) are added. If the LRT is not statistically significant, this means that the second variable is conditionally independent on the disease, given the first one.

#### Studying the association between SNPs in the *HLA* region and MS

The lack of SNP genotypes on all the individuals who are genotyped for *HLA* loci (see supplementary methods) led us to adopt a method that uses *HLA* loci as proxies in the association analysis in order to impute the missing SNPs. We performed the analysis using UNPHASED software [46].

This method estimates haplotype frequencies across all specified markers, including the SNPs of interest and the tag *HLA* alleles. However the model for disease risk includes an effect only for the SNPs of interest. When the data include some individuals with genotypes for all *HLA* and SNP markers, and others with genotypes for the *HLA* alleles only, the method uses a missing data likelihood to estimate the association for the SNPs of interest, using information from the *HLA* alleles when the SNP genotype is missing.

In order to distinguish the association of SNPs from their linkage disequilibrium with association *HLA* alleles, we performed a conditional analysis allowing for the *HLA* association. Here the disease model includes effects for both the SNPs of interest and the *HLA* alleles, but only the SNP effects are tested [47]. Again, a missing data likelihood is used to allow for both haplotype phase uncertainty and missing SNP data.

## Results

Structure of the family nuclei, % of missing genotype, number of individuals genotyped for both *HLA* and SNPs in term of the structure of the nuclei are fully described in the supplementary material.

### Analysis of the ancestral haplotypes

The ancestral haplotypes reconstruction in the Nuoro population shows that the *A2Cw7B58DR2DQ1* haplotype has a frequency of 4.3% while the *A30Cw5B18DR3DQ2* haplotype has a frequency of 15.2%. The frequencies of the alleles at each locus for every ancestral haplotype are reported in the supporting material S3. On the basis of a multiplicative model, the effect of the first haplotype versus all the others is protective, OR 0.27(95% Confidence Interval CI 0.13–0.57), while that of the second is deleterious, OR 1.78 (95% CI 1.26–2.50).

As to the effect model, the allele *B18* is the only one showing a statistically significant departure from a multiplicative model (p = 0.006). The recessive model is the best fitting model (p = 0.0001) on the basis of the Wald test. The empirical p-values of the single locus marginal test show that for Class I loci, in the protective haplotype, only *Cw* and *B* are significantly associated to MS, while in the deleterious haplotype *A, Cw*, *B* are all associated to the disease. For Class II loci, *DR* and *DQ* are significantly associated with the disease both for the protective and the deleterious haplotypes (Table 1).

**Table 1:** *A2Cw7B58DR2DQ1* protective haplotype and *A30Cw5B18DR3DQ2* deleterious haplotype: genotype frequency of the allele composing the haplotypes in pseudocases, pseudocontrols, Odds ratio (OR) and its 95% Confidence Interval (CI), p-value of the Wald's Test of association and empirical p-value computed under the multiplicative model.

Locus Genotypes	Pseudocases N	Pseudocontrols N	OR	95% CI		LRT p	REG p		LRT Empirical p		
**PROTECTIVE HAPLOTYPE: ** ***A2Cw7B58DR2DQ1***											
***A***						1.00E-01		9.90E-02		1.00E-01	
***X/X***	117	98									
***X/2***	72	91	0.80	0.63–1.04							
***2/2***	19	19	0.65	0.39–1.08							
***Cw***						7.10E-01		1.80E-02		3.80E-02	
***X/X***	120.63	103.6									
***X/7***	64	68.57	0.76	0.61–0.95							
***7/7***	23.36	35.86	0.58	0.37–0.91							
***B***						4.90E-01		1.30E-05		1.30E-05	
***X/X***	193	168									
***X/58***	15	35.66	0.33	0.20–0.55							
***58/58***	0	4.34	0.11	0.04–0.30							
***DR***						3.90E-01		9.25E-05		9.00E-05	
***X/X***	162	135.5									
***X/2***	44	63.34	0.54	0.39–0.73							
***2/2***	2	9.19	0.28	0.15–0.54							
***DQ***						2.00E-01		1.02E-07		1.00E-07	
***X/X***	128	91.14									
***X/1***	72	87.81	0.5	0.39–0.65							
***1/1***	8	29.05	0.26	0.15–0.42							
**DELETERIOUS HAPLOTYPE: ** ***A30Cw5B18DR3DQ2***											
***A***					2.20E-01			2.00E-03			1.20E-03
***X/X***	102	131									
***X/30***	86	62	1.54	1.17–2.03							
***30/30***	17	12	2.37	1.36–4.10							
***Cw***					2.50E-01			2.00E-03			1.26E-03
***X/X***	97.02	125.86									
***X/5***	81.02	60.65	1.49	1.16–1.91							
***5/5***	26.96	18.49	2.20	1.33–3.65							
***B***					6.00E-03			1.80E-05			1.78E-05
***X/X & X/18***	170	195									
***18/18***	35	10	4.01	2.13–7.58							
***DR***					9.50E-01			1.00E-03			1.30E-03
***X/X***	94	119.7									
***X/3***	80	67.5	1.50	1.17–1.91							
***3/3***	31	17.8	2.34	1.37–3.64							
***DQ***					2.40E-01			1.00E-03			9.20E-04
***X/X***	74	104.14									
***X/2***	98	77.90	1.51	1.18–1.93							
***2/2***	33	22.96	2.28	1.40–3.72							

**LRT p** = p-value of the Likelihood Ratio Test for testing departure from a multiplicative model;

**REG p** = unadjusted p-value of an unconditional logistic regression;

**EMPIRICAL p** = empirical p-value of the logistic regression obtained after permutation;

**X** refers to all alleles other than the one taken into consideration.

We performed an analysis which aims at identifying which loci composing the deleterious and protective haplotype, can solely explain the association with the disease. We carried out on the *protective* and the *deleterious* haplotype separately considered a sole variant and independent test and a conditional independence test to investigate which loci within Class I and Class II could be considered as a single variant associated with the disease (Table 2).

**Table 2:** Sole variant and independent test carried out within the Class I and Class II loci of both protective and deleterious haplotype.

**CLASS I LOCI-Sole variant and independent test**							
	**Protective Ancestral Haplotype**				**Deleterious Ancestral Haplotype**		
**Test**	Model 1		Model 2	LRT Empirical p	Model 1	Model 2	LRT Empirical p
Sole Variant*	*A2+Cw7+B58*		*B58*	8.5E-1	*A30+Cw5+B18*	*B18*	4.0E-1
Independent**	*A2+Cw7+B58*		*A2+Cw7*	4.0 E-4	*A30+Cw5+B18*	*A30+Cw5*	5.0E-4
**CLASS II LOCI- Sole variant test and independent test**							
		**Protective Ancestral Haplotype**			**Deleterious Ancestral Haplotype**		
**Test**		Model 1	Model 2	LRT Empirical p	Model 1	Model 2	LRT Empirical p
Sole Variant*		*DR2+DQ1*	*DQ1*	7.4E-1	*DR3+DQ2*	*DQ2*	4.9E-1
Independent**		*DR2+DQ1*	*DR2*	1.0E-3	*DR3+DQ2*	*DR3*	2.9E-1

**LRT EMPIRICAL p** = empirical p-value of the likelihood ratio test obtained after permutation; ^*^comparison of a model including both ‘everything else’ and the ‘particular variant’ with a model including the ‘particular variant’. If the p-value for the LRT is not statistically significant the particular variant is the ‘sole variant’. ^**^Comparison of a model in which ‘everything else’ and the ‘particular variant’ are included with a model including ‘everything else’. If a p-value for the LRT is statistically significant, then this particular variant is associated independently on everything else.

For the *protective* haplotype in Class I, *B58* is a sole variant associated with MS; on the contrary, from the independence test B58 turns out to be independently associated with MS given *A2 Cw7* (p-value of 0.0004 of the LRT).

In Class II, the sole variant is *DQ1*(empirical p-value of 0.74) and also *DQ1* is independently associated with MS given *DR2* (p-value = 0.001).

As for the *deleterious* haplotype, on the basis of the LRT we identify B18 as being the sole variant in Class I loci. *B18* is also independently associated with MS given *A30 Cw5*. As for the *deleterious* haplotype, *DR2* turns out to be a sole variant, but it is not independently associated to MS. The strong linkage disequilibrium between the *DR3* and *DQ2* alleles (D' = 0.98, R^2^ = 0.75) does not allow us to identify which of the two loci is responsible of the association (Table 2).

To further pinpoint the specific allele contributing to MS susceptibility, a conditional independent test was carried out between the sole variants identified within Class I loci and the sole variant within Class II loci.

For the *protective* haplotype, *DQ1* is not conditionally independent on MS given *B58* and *B58* viceversa. For the *deleterious* haplotype, the *DR3* locus appears to be conditionally independent on the disease given the *B18* locus, while *DR3* is not conditionally independent on MS given *B58* (Table 3).

**Table 3:** Conditional Independence Test: comparison of the model including both variants and the model including just one of the sole variants within the deleterious and the protective haplotype.

Conditional independence test					
Protective Ancestral Haplotype			Deleterious Ancestral Haplotype		
Model 1	Model 2	LRT Empirical p	Model 1	Model 2	LRT Empirical p
*B58+DQ1*	*B58*	9.7 E-5	*B18+DR3*	*B18*	1.9E-1
*B58+DQ1*	*DQ1*	7.7 E-4	*B18+DR3*	*DR3*	1.0E-4

**LRT EMPIRICAL p** = empirical p-value of the likelihood ratio test obtained after permutation.

Within each haplotype, we also estimated the effect in terms of odds ratio of each sole variant in Class I adjusted for the effect of the sole variant in Class II loci versus all the other variants in Class I also adjusted for the effect of the sole variant in Class II loci, by fitting a model including both the sole variant in Class I and the sole variant in Class II.

In agreement with the conditional independent tests, within the protective haplotype, both the loci *B58* and *DQ1* show a strong protective statistically significant effect while within the deleterious haplotype only the *B18* locus show a statistically significant deleterious effect. This further confirms that the *DR3* locus is conditionally independent on the disease given the *B18* locus (for details see supporting material S4).

### Investigating the effect of all alleles at the *B*, *DR* and DQ loci

We investigated the effect of the complete available set of alleles for *B*, *DR*, *DQ* loci and only alleles belonging to the ancestral haplotypes turned out to be significant (Table 4).

**Table 4:** Association between *B*, *DR* and *DQ* loci with MS.

Locus	Alleles	Pseudocases	Pseudocontrols	REG Empirical p
*B*	*5*	32	36	NS
	°	81	76	NS
	*12*	25	22	NS
	*14*	28	32	NS
	***18***	**159**	**112**	**6.74E-04**
	*21*	38	38	NS
	*22*	8	13	NS
	*35*	39	51	NS
	***58***	**16**	**46**	**1.93E-04**
*All B alleles*	***-***	**-**	**-**	**1.8E-04**
*DR*	*1*	22	23.30	NS
	***2***	**50**	**84.80**	**1.93E-04**
	***3***	**150**	**108.00**	**5.78E-04**
	*4*	78	71.30	NS
	*5*	58	52.50	NS
	*6*	24	39.80	NS
	*7*	18	25.30	NS
	*8*	21	9.72	NS
	*10*	5	11.60	NS
*All DR alleles*				**2.33E-07**
*DQ*	***1***	**92**	**152**	**7.00E-09**
	***2***	**173**	**131**	**3.85E-04**
	*3*	141	133.0	NS
	*4*	20	10.5	NS
*All DQ alleles*				2.72E-08

The table reports the number of pseudocases and pseudocontrols and empirical p-value of the alleles. Alleles are tested against all other alleles in the same class B° contains the alleles (*7,8,13,15,16,27,37,40*) that have a frequency<4%.

**REG Empirical p** = empirical p-value of the logistic regression obtained after permutation.

**NS** = Not Significant.

Since a strong association with an allele (for example protective) can mask the association with another allele whose effect is protective as well, we investigated the effect of the alleles other than those composing the ancestral haplotypes by performing an analysis that conditioned on a specific allele belonging to the ancestral haplotype. Details on the conditioning method are given in the supporting material S5. For the *B* locus, the effect of *B58* emerges only after we condition on *B18* and no other allele is significant; for the *DR* locus, *DR6* is significant, with a protective effect, when conditioning on *DR2* only and when conditioning on both *DR2* and *DR3*. Finally, conditioning on *DQ2* (deleterious allele) makes the deleterious association effect with both *DQ3* and *DQ4* to be detected. *DQ1* remains significant also when conditioning on *DQ2* (supporting material S5).

### SNP analysis

We now report the results of our exploratory analysis using a nominal significance level p<0.005 for unconditional tests. We studied the association between MS and 796 SNPs by using the program UNPHASED with the tag and missing options. We used the tag option to overcome the discrepancy between the individuals typed for *HLA* loci and the individuals typed for SNPs, the latter being much less numerous than those typed for *HLA*. We specified as tag markers the *HLA* loci composing the ancestral haplotypes which did not show a significant association with MS after conditioning on the most strongly associated loci. More specifically, we performed two analyses: (1) where tag markers are *HLA* loci composing the protective ancestral haplotype (*A2*,*Cw7*,*DR2*) and (2) where tag markers are *HLA* loci composing the deleterious ancestral haplotype (*A30*,*Cw5*,*DQ2*). This choice was motivated by two considerations (1) we did not want to influence the association test for SNPs by choosing as tags the SNPs which turned out significantly associated with MS (2) we could not exclude that some SNPs associated with disease might be in LD with the protective alleles and some in LD with the deleterious allele. In each case we recoded each of the *HLA* loci into a binary variable, indicating the presence of the allele present on the extended protective (or deleterious) haplotype.

The analysis using the protective haplotype to predict missing SNPs leads to signals of association with SNPs located in the following genes: *PSORS1C1* (Psoriasis susceptibility 1 candidate 1 - ENSG00000204540), *TCF19* (Transcription factor 19 - ENSG00000137310), *BAT2* (*HLA-B* associated transcript 2 - ENSG00000204469), *BAT3* (*HLA-B* associated transcript 3 - ENSG00000204463), *NOTCH4* (Neurogenic locus notch homolog protein 4 - ENSG00000204301), *ITPR3* (Inositol 1,4,5-triphosphate receptor, type 3 - ENSG00000096433) and *PACSIN1* (Protein kinase C and casein kinase substrate in neurons 1 - ENSG00000124507).

The analysis using the deleterious haplotype to predict missing SNPs leads to highlighting the same set of genes as the analysis with the protective haplotype, and in addition *BTNL2* (Butyrophilin-like 2 - ENSG00000204290), *BAK1* (BCL2-antagonist/killer 1 - ENSG00000030110) and *HLA*-*DOB* (Major histocompatibility complex, class II, DO beta - ENSG00000204273), *TAP2* (Transporter 2, *ATP*-binding cassette, sub-family B - ENSG00000204267). The p-values of the null hypothesis of association are reported in the supporting material S6.

We finally tested whether the effect seen in these SNPs was due to the LD with the associated *HLA* alleles. For this purpose we performed an analysis of the associated SNPs, conditioning on the alleles of the protective and deleterious haplotypes which are primarily associated (see the paragraph “Analysis of the ancestral haplotypes”). For this analysis we used UNPHASED with the condition option together with the tag options just described. Conditioning on the *B58* locus does not significantly change the SNP specific p-value, whereas conditioning on *DQ* does and some SNPs become not significantly associated with MS. After conditioning, the genes, in which the statistically significant SNPs are located, are *NOTCH4*, *BTNL2*, *ITPR3* and *PACSIN1 HLA*-*DOB* and *TAP2* (supporting material S7).

## Discussion

We carried out a family based association study to further investigate the role of *HLA* region (on Chromosome 6p21) in MS susceptibility. The contribution of *HLA* genes in MS pathogenesis is well established. Nowadays the association of MS with the *HLA*-*DR2* haplotype, comprising the *HLA*-DRB1*1501 and *HLA*-DRB5*0101 alleles, is the most replicated result among very different populations and a biological explanation was recently found for the strict co-occurrence of these alleles. *DR2b* protein (encoded by *HLA*-DRB1*1501) in fact promotes a strong immune response, while *DR2a* protein (encoded by *HLA*-DRB5*0101) has an opposite and regulatory effect, inducing apoptosis of T cells. Both proteins participate in the immune response modulation and their interaction is a typical example of epistasis [48]. We choose to analyse the isolated population of Nuoro, Sardinia, which is characterised by a very high prevalence of MS (4–5 times higher than the Italian mainland) and by a small number of very frequent *HLA* haplotypes. The two most frequent *HLA* haplotypes are *A30Cw5B18DR3DQ2* (14.6%) and *A2Cw7B58DR2DQ1* (6%) which for their frequency are considered ancestral. To date, research on MS in Sardinia has been mainly focusing on the most frequent *HLA* ancestral haplotype and only on loci *A, B, DR* [36]–[38]. We have gone a step forward and collected information also on the *Cw* and *DQ* loci and hence we were able to consider the “entire” haplotype from A to DQ. We studied both *A30Cw5B18DR3DQ2* and *A2Cw7B58DR2DQ1*.

We were able to refine Bitti's results [37] as we found that *A30Cw5B18DR3DQ2* increases the risk of MS. *B18* is confirmed to be the most predictive single locus haplotype among Class I loci, while for Class II both *DR3* and *DQ2* are positively associated: however we can't differentiate between them as they are in a strong LD with each other.

Moreover, we found the second most frequent haplotype *A2Cw7B58DR2DQ1* to be protective. This result represents an interesting novelty regarding the association between the *HLA* alleles and MS. Locus *B* (*B58*) is again the most significant single allele among Class I loci, while *DQ1* among Class II loci.

As both Class I and Class II loci turned out to be associated with MS, we based our subsequent analysis on investigating whether they are associated independently. We confirmed previous results [31], [49] of Class I loci having an independent effect on the risk of developing MS. Indeed our findings seem to support the claim that both Class I and Class II loci have an effect on MS susceptibility, but that they act independently from each other. The signal we found for Class I loci still exist even when conditioning on Class II loci and when taking LD into account. From a biological perspective this makes sense, as *HLA* Class II molecules are involved in the triggering of adaptive immune response and are expressed by Antigen Presenting Cells (APC), while *HLA* Class I typically interact with cytotoxic CD8+T cells and are expressed by all nucleated cells. CD8+T cells are of central importance in lesion pathogenesis as they outnumber CD4+T cells in MS lesions [46]. Moreover, *HLA* Class I molecules are ligand for the KIR (Killer cell immunoglobulin-like receptors) protein family, expressed by Natural Killer (NK) cells in different combinations and amounts[50]. The role of NK cells in MS pathogenesis is controversial as they promote both deleterious and protective effects on neuronal cells[51]. Not much is known on the biological function of KIR receptors and for now no direct connection has been seen between *KIR* genes and MS. However, they cluster on Chromosome 19q13.4, a region previously found in linkage studies to be associated with MS[52].

To further enrich our study, we finally performed an exploratory analysis on a set of 796 SNPs in the same *HLA* region. Genomic regions containing SNPs associated with MS were investigated using Ensembl genome browser Release 50 at www.ensembl.org and HapMap - Phase 3 release data at www.hapmap.org. The bioinformatic screen showed that the majority of SNPs lie in genes with biological functions related to immune response or to neuronal signal transmission. This analysis does not add conclusive evidence in favour of any of these genes, given its exploratory nature, however *NOTCH4*, *BTNL2* and *BAK1* deserve some remarks. The *NOTCH* gene family participates in the control of myelination and T cells commitment. Previous studies indicate that developmental signal transduction pathways, involving *NOTCH1* (Neurogenic locus notch homolog protein 1)activation, are re-expressed in damaged neurons in MS. The effect of this signalling is the inhibition of remyelination, which is restored after *NOTCH1* inhibition [53]. Besides, *NOTCH1* controls the differentiation of naïve T CD4+cells into T helper type 1 (Th1) cells. Th1 lymphocytes have a central role in immune response towards intracellular pathogens and their aberrant activity has been associated to the induction of certain autoimmune diseases, MS included [54].

*NOTCH1* directly inhibits the expression of T-box transcription factor TBX21, that encodes a transcription factor necessary to Th1 maturation [55]: as a consequence, Th1 maturation is inhibited [56]. *NOTCH* proteins are thus interesting because their inhibition should provide a double beneficial effect against MS, promoting remyelination and decreasing the number of Th1 cells. *BTNL2* is a negative regulator of T cell proliferation. Some variants of *BTNL2* and *NOTCH4*, different from those here tested, have been found associated with MS in two studies, but in both cases the association observed was considered secondary to *HLA DR15* influence [57], [58]. *BAK1* accelerates apoptosis, contrasting the antiapoptotic molecule B-cell CLL/lymphoma 2 (*BCL2*). Apoptosis is an important feature in MS pathogenesis. Deregulation of this process can in fact promote both the survival of an excessive number of auto-reactive immune cells and the death of oligodendrocytes [59]. Nevertheless, apoptosis is necessary to physiological oligodendrocytes maturation [60].

However, as this was just an exploratory analysis, with many untyped data which were imputed conditioning on *HLA* loci, these last results need to be confirmed both on our population and replicated in others. Still, it appears that studying *HLA* main loci only could not be enough as other *HLA* genes may be involved and this could open new perspectives on the study of MS. In this perspective, Sardinia and especially the Nuoro population provide an important contribution. Indeed, the general population of Sardinia has been shown to have similar levels of LD to outbred populations, while isolated villages within Sardinia have substantially increased levels of LD compared to cosmopolitan populations. The province of Nuoro is one of the oldest isolates founded over 3000 years ago. The subsequent isolation of this area has likely contributed to its divergence from the other European populations in terms of allele frequencies, yet its age has afforded many opportunities for recombination to occur between markers. Service et al. [61] suggest that, considering the length of the LD maps only, the association analyses in samples from Sardinia would require at least 30% fewer markers than studies in outbred population. For this reason, in this first part of the study LD helps us to identify, more easily, the region which contains the genes associated with MS but fine mapping is harder as a result and the power of our conditional tests is affected by the strong LD in this population.

## Supporting Information

Supporting Material S1 Ancestral haplotypes.
(0.03 MB DOC)

Supporting Material S2 HLA typing, SNP typing and quality control; % Call rate; SNP genotyping.
(0.04 MB DOC)

Supporting Material S3 Frequency of A2Cw7B58DR2DQ1 and of A30Cw5B18DR3DQ2 haplotypes and their composing Class I and Class II alleles in pseudocases, pseudocontrols and in the whole population.
(0.04 MB DOC)

Supporting Material S4 Effect of Class I locus adjusted for the effect of the Class II locus, OR and its 95% CI.
(0.03 MB DOC)

Supporting Material S5 Empirical p-value for the null hypothesis of association for the alleles at the B,DR and DQ loci after conditioning for ancestral haplotypes alleles.
(0.04 MB DOC)

Supporting Material S6 MS and SNPs with tag markers alleles composing the protective ancestral haplotypes: significant findings reported.
(0.04 MB DOC)

Supporting Material S7 MS and SNPs with tag markers alleles composing the deleterious ancestral haplotypes: significant findings reported.
(0.05 MB DOC)
