# Supplementary material for: Association between Protective and Deleterious HLA Alleles with Multiple Sclerosis in Central East Sardinia
Source: PLoS One. 2009 Aug 5;4(8):e6526. doi: 10.1371/journal.pone.0006526 (PMC2716537; doi:10.1371/journal.pone.0006526)
Supplement: Supporting Material S1 — Ancestral haplotypes. (0.03 MB DOC) [file pone.0006526.s001.doc]

**Supplementary material S1.**

**Ancestral haplotypes**

Historically speaking the term *ancestral haplotype* was coined in the 80s, when people started realising that the word *haplotype* was widely misused and it was agreed to use the term *supratype* for combinations of alleles that could reflect haplotypes, but that were defined by the combination rather than by the pattern of segregation within a family^i^.

Shortly afterwards, it turned out that most supratypes of interest were carried on one haplotype, as the component alleles were found to be in positive linkage disequilibrium in population studies^ii^. As it was difficult to imagine how alleles could be maintained in positive linkage disequilibrium over many generations unless the haplotype was conserved *en bloc*^iii^, the term ancestral haplotype was introduced to define this haplotype of large ancestral families.

As already mentioned the Sardinian area of Nuoro is a genetically well defined area, with a high rate of endogamy and with a low rate of migration and it is thus characterised by a small number of very frequent haplotypes^iv^. Seven of them –*A1Cw7B49DR4*; *A2Cw5B18DR3*; *A2Cw7B58DR2*; *A11Cw4B35DR1*; *A30Cw5B18DR3*; *A33Cw8B14DR1*; *A2Cw7B18DR5*- have a frequency higher than 0.85%^v^ and thus are ancestral haplotypes. The latter has also been reported among the Ashkenazi Jews^vi^. Another two haplotypes or combinations of them have also been found in LD in other Caucasoid populations though with a much lower frequency than in Sardinia: the *A1Cw7B49DR4* in Spain^vii^ and the *A30Cw5B18DR3* in the Basque population^viii^, in Spain^vii,ix^ and also among the Algerian Berbers^x^.

None of these seven haplotypes was found in LD in the Negroid or Mongoloid populations^xi,xii^. These findings suggest that the Sardinian population is the result of a superposition on a paleo-Mediterrenean substratum of different ethnic groups, largely composed by Caucasoid from Central Italy and the European regions of the North West Mediterrenean basin (ancestral haplotypes *A1Cw7B8DR3*, *A3Cw7B7DR2*; *A11Cw4B35DR5*), but also from North Africa and the South Eastern regions of the Mediterrenean basin (ancestral haplotypes: *A33Cw8B65DR1*; *A30Cw6B13DR7*)^xiii^.

**Supplementary References**

Dawkins RL (1982) Muscoloskeletal disease and D-Penicilllamine: concepts and models. In: Dawkins RL, Christiansen FT, Zilko PJ, editors. Muscoloskeletal Disease and D-Penicillamine. Amsterdam: Excerpa Medica. pp. 1.

Degli-Esposti MA, Leaver AL, Christiansen FT, Witt CS, Abraham LJ, Dawkins RL (1992) Ancestral haplotypes: conserved population MHC haplotypes. Hum Immunol 34: 242-252.

Dawkins RL, Christiansen FT, Kay PH, Garlepp M, McCluskey J, Hollingsworth PN, Zilko PJ (1983) Disease associations with complotypes, supratypes and haplotypes. Immunol Rev 70: 1-22.

Bitti PP, Murgia BS, Ticca A, Ferrai R, Musu L, Piras ML, Puledda E, Campo S, Durando S, Montomoli C, Clayton DG, Mander AP, Bernardinelli L (2001) Association between the ancestral haplotype HLA A30B18DR3 and multiple sclerosis in central Sardinia. Genet Epidemiol 20: 271-283.

Contu L, Arras M, Carcassi C, La Nasa G, Mulargia M (1992) HLA structure of the Sardinian population: a haplotype study of 551 families. Tissue Antigens 40: 165-174.

Cohen N, Brautbar C, Font MP, Dausset J, Cohen D (1986) HLA-DR2-associated Dw subtypes correlate with RFLP clusters: most DR2 IDDM patients belong to one of these clusters. Immunogenetics 23: 84-89.

Segurado OG, Giles CM, Iglesias-Casarrubios P, Corell A, Martinez-Laso J, Vicario JL, Arnaiz-Villena A (1991) C4 Chido 3 and 6 distinguish two diabetogenic haplotypes: HLA-B49, SC01,DR4,DQw8 and B8,SC01,DR3,DQw2. Immunobiology 183: 12-22.

Cambon-de Mouzon A, Ohayon E, Hauptmann G, Sevin A, Abbal M, Sommer E, Vergnes H, Ducos J (1982) HLA-A, B, C, DR antigens, Bf, C4 and glyoxalase I (GLO) polymorphisms in French Basques with insulin-dependent diabetes mellitus (IDDM). Tissue Antigens 19: 366-379.

Regueiro JR, Arnaiz-Villena A (1988) Human MHC class III (Bf, C2, C4) genes and GLO: their association with other HLA antigens and extended haplotypes in the Spanish population. Tissue Antigens 31: 14-25.

Bouali M, Dehay C, Benajam A, Poirier JC, Degos L, Marcelli-Barge A (1981) HLA-A,B,C, Bf and glyoxalase I polymorphisms in a sample of the Kabyle population (Algeria). Tissue Antigens 17: 501-506.

Tusji K, Aizawa M, Sasazuki T (1991) Allele and haplotype frequencies for selected ethnic groups. Data Analysis Book II 443-542.

Baur MP, Neugebauer M, Albert ED (1984) Reference tables of three-locus haplotype frequencies and delta values in Caucasians, Orientals and Negroids. In: Albert ED, Baur MP, Mayr WR, editors. Histocompatibility testing. Heidelberg: Springer-Verla. pp. 756-760.

Contu D, Carcassi C, Trucco M (1991) Diabetes susceptibility in Sardinia. Lancet 338: 65.
