# Supplementary material for: Association between Protective and Deleterious HLA Alleles with Multiple Sclerosis in Central East Sardinia
Source: PLoS One. 2009 Aug 5;4(8):e6526. doi: 10.1371/journal.pone.0006526 (PMC2716537; doi:10.1371/journal.pone.0006526)
Supplement: Supporting Material S2 — HLA typing, SNP typing and quality control; % Call rate; SNP genotyping. (0.04 MB DOC) [file pone.0006526.s002.doc]

**Supplementary material S2.**

**DNA extraction, *HLA* typing, SNP typing and quality control**

Buffy coat preparation and DNA extraction were performed following a classical salting out protocol. Further DNA extraction from buffy coat and gene sequencing were performed at the Centre National de Genotypage, Evry, France.

All family members were typed for the *HLA*-A, Cw, B, DR and DQ antigens and relative splits using Micro-lymphocyte cytotoxicity test (LCT).

Some people were potentially homozygous on some *HLA* locus from the serological typing. To verify the homozygosity, we performed genomic typing at those particular loci using the DynaI RELI^TM^SSO typing kit provided by Invitrogen.

Mendelian consistency was tested with the software Progeny. We also calculated Hardy-Weinberg Equilibrium (HWE) on controls (p-value ≥0.01). The Cw locus was not in HWE, but this locus did not show anyhow any evidence of significance.

% of samples with non-missing data over the total number of genotyped samples are shown in the table below,

**Table % Call rate**

| ***HLA* locus** | **%** |
| --- | --- |
| A | 100 |
| Cw | 95.54 |
| B | 99.84 |
| DR | 92.20 |
| DQ | 87.74 |

Of the 628 people, 214 individuals were genotyped for both *HLA* and SNPs: 117 are controls (49.57 % parents, 22.22% partner, 28.21 siblings) and 97 cases (34.02% cases of trio 1, 14.43% of trio 2 and 51.55% are unrelated cases).

**SNP genotyping**

SNPs were genotyped at Centre National de Genotypage, Evry, France using a GoldenGate Custom Panel of 1536 assays. SNPs meeting the following criteria were then selected for subsequent analyses:

1. a minor allele frequency in founders greater than 5%.
2. a minimum call rate (number of reactions with data available over the total number of genotyped persons) of 90% on the whole sample (that is equivalent to say a number of missing ≤ 10%)
3. consistency with Hardy-Weinberg Equilibrium in founders at a p-value ≥ 0.01
4. heterozygosity in founders greater than 0.25
5. presence of homozygous genotypes of major/minor alleles and presence of the heterozygous genotype

796 SNPs met the mentioned standard criteria.
