# Supplementary material for: Association between Protective and Deleterious HLA Alleles with Multiple Sclerosis in Central East Sardinia
Source: PLoS One. 2009 Aug 5;4(8):e6526. doi: 10.1371/journal.pone.0006526 (PMC2716537; doi:10.1371/journal.pone.0006526)
Supplement: Supporting Material S3 — Frequency of A2Cw7B58DR2DQ1 and of A30Cw5B18DR3DQ2 haplotypes and their composing Class I and Class II alleles in pseudocases, pseudocontrols and in the whole population. (0.04 MB DOC) [file pone.0006526.s003.doc]

**Supplementary material S3**.

**Table.** Frequency of *A2Cw7B58DR2DQ1* and of *A30Cw5B18DR3DQ2* haplotypes and their composing Class I and Class II alleles in pseudocases, pseudocontrols and in the whole population .

| **Protective: *A2Cw7B58DR2DQ1*** | | | | | | |
| --- | --- | --- | --- | --- | --- | --- |
|  | **Classe I** | | | **Classe II** | |  |
| Locus/allele | ***A2*** | ***Cw7*** | ***B58*** | ***DR2*** | ***DQ1*** | **HAPLO** |
| Pseudocases (n=416) | 110 (26.5%) | 110 (26.5%) | 15 (3.6%) | 48 (11.5%) | 88 (21.15%) | 7.89 1.9%) |
| Pseudocontrols (n=416) | 129 (31%) | 140 (33.65%) | 44.3 (10.6%) | 81.7 (19.6%) | 146 (35%) | 27.6 6.6%) |
| All | 28.7% | 30% | 7% | 15.6% | 28% | 4.3% |
| **Deleterious: *A30Cw5B18DR3DQ2*** | | | | | | |
|  | **Classe I** | | | **Classe II** | |  |
| Locus/allele | ***A30*** | ***Cw5*** | ***B18*** | ***DR3*** | ***DQ2*** | **HAPLO** |
| Pseudocases (n=416) | 120 (28.8%) | 135 (32.5%) | 153 (36.8%) | 142 (34%) | 164 (39.5%) | 78.8 (19%) |
| Pseudocontrols (n=416) | 86 (20.7%) | 97.6 (23.5%) | 106 (25.5%) | 103 (24.75%) | 124 (29.8%) | 47.9 (11.5%) |
| All | 24.7% | 28% | 31% | 29.5% | 34.6% | 15.2% |
