# Supplementary material for: Association between Protective and Deleterious HLA Alleles with Multiple Sclerosis in Central East Sardinia
Source: PLoS One. 2009 Aug 5;4(8):e6526. doi: 10.1371/journal.pone.0006526 (PMC2716537; doi:10.1371/journal.pone.0006526)
Supplement: Supporting Material S4 — Effect of Class I locus adjusted for the effect of the Class II locus, OR and its 95% CI. (0.03 MB DOC) [file pone.0006526.s004.doc]

**Supplementary material S4.**

**Table.** Effect of Class I locus adjusted for the effect of the Class II locus, OR and its 95% CI within the protective and deleterious haplotype

| **Marker** | **Model** | **0R** | **96%CI** |
| --- | --- | --- | --- |
| **Protective Ancestral Haplotype** | | | |
| *DQ1* | *B58+DQ1* | 0.57 | 0.44-0.73 |
| *B58* | *B58+DQ1* | 0.42 | 0.25-0.70 |
|  |  |  |  |
| **Deleterious Ancestral Haplotype** | | | |
| *DR3* | *B18+DR3* | 1.20 | 0.92-1.57 |
| *B18* | *B18+DR3* | 1.83 | 1.30-2.57 |
