# Supplementary material for: Association between Protective and Deleterious HLA Alleles with Multiple Sclerosis in Central East Sardinia
Source: PLoS One. 2009 Aug 5;4(8):e6526. doi: 10.1371/journal.pone.0006526 (PMC2716537; doi:10.1371/journal.pone.0006526)
Supplement: Supporting Material S5 — Empirical p-value for the null hypothesis of association for the alleles at the B,DR and DQ loci after conditioning for ancestral haplotypes alleles. (0.04 MB DOC) [file pone.0006526.s005.doc]

**Supplementary material S5**

**Table.** Empirical p-value for the null hypothesis of association for the alleles at the *B*,*DR* and *DQ* loci after conditioning for ancestral haplotypes alleles.

The idea of conditioning arises from the consideration that a strong association with an allele (for example protective) can mask the association with another allele whose effect is protective as well. We repeated the same procedure for each allele in turn composing the ancestral haplotypes. The analysis is performed via a regression model applied to the data set in which all the configurations with the *DR2* allele, for example, have been eliminated from the data set and the remaining have been rescaled. As described above each “nucleus” provide 2 transmitted and 2 untransmitted chromosomes and the sum of the weights within each nucleus should hence be 4. The rescaling algorithm rescales the weight of the configuration carrying an allele different from *DR2* in such a way the sum of the weights within each nucleus remains equal to 4. This is obtained by dividing the weight of each allele by the sum of the weights of the rest of alleles (excluding that on which we condition on) and multiple by 2. This is done separately within the transmitted/untransmitted configurations within each nucleus. The permutation scheme embedded in the regression approach allows to correct for multiple testing arising from the fact that at each locus we observed many alleles.

| **Locus** | **Conditioning alleles** | | |
| --- | --- | --- | --- |
| ***B*** | *B18 (+)* | *B58 (-)* | *B18* and *58* |
| ***18*** | - | NS | - |
| ***58*** | 2E-03 (-) | - | - |
|  |  |  |  |
| ***DR*** | DR_2_ (-) | DR_3_ (+) | DR_2_ and DR_3_ |
| ***2*** | - | 0.015(-) | - |
| ***3*** | - | - | - |
| ***6*** | 9E-04(-) | NS | 1.4E-02(-) |
|  |  |  |  |
| ***DQ*** | DQ_1_ (-) | DQ_2_ (+) | DQ_1_and DQ_2_ |
| ***1*** | - | 5E-06(-) | - |
| ***2*** | NS | - | - |
| ***3*** | NS | 2E-03(+) | NS |
| ***4*** | NS | 2E-02(+) | NS |

- =not applicable being the conditioning allele

NS = Not Significant

*alleles with a frequency lower than 4%

(+) positive association (deleterious effect)

(-) negative association (protective effect)
