# Supplementary material for: Association between Protective and Deleterious HLA Alleles with Multiple Sclerosis in Central East Sardinia
Source: PLoS One. 2009 Aug 5;4(8):e6526. doi: 10.1371/journal.pone.0006526 (PMC2716537; doi:10.1371/journal.pone.0006526)
Supplement: Supporting Material S6 — MS and SNPs with tag markers alleles composing the protective ancestral haplotypes: significant findings reported. (0.04 MB DOC) [file pone.0006526.s006.doc]

**Supplementary material S6.**

**Table.** MS and SNPs with tag markers alleles composing the protective ancestral haplotypes: significant findings reported (p<0.005). For each SNP found to be associated with MS while using as tag SNPs the alleles composing the protective haplotype, we show the p-value obtained under the multiplicative model before and after conditioning on *B58* and *DQ1*. The last column reports the gene in which the SNP is located, the number in () indicates the location in the gene. Where the location is not uniquely defined more than one gene is reported

| **SNP** | **Distance** | **P-value**  **before conditioning** | **Conditioning**  **on *B58*** | **Conditioning on *DQ1*** | **Gene** |
| --- | --- | --- | --- | --- | --- |
| rs2535310 | 31161246 | 2.40E-03 | 2.30E-02 | 3.09E-01 | none |
| rs2233966 | 31188838 | 1.60E-03 | 1.00E-02 | 1.70E-01 | *C6orf15(u) CDSN(d) PSORS1C1(u)* |
| rs2073719 | 31220904 | 2.06E-03 | 3.70E-03 | 9.70E-02 | *CCHCR1(i)* |
| rs9263758 | 31223853 | 2.30E-03 | 5.00E-03 | 9.70E-02 | *CCHCR1(i)* |
| rs2073723 | 31238057 | 2.20E-03 | 3.60E-03 | 8.00E-02 | *CCHCR1(u) POU5F1(u) TCF19(i)* |
| ***HLA* *B*** | 31429628-31433001 | - | - | - | ***HLA B*** |
| rs384247 | 32292552 | 1.80E-03 | 4.30E-03 | 5.70E-02 | *NOTCH4(i)* |
| rs715299 | 32297819 | 1.60E-03 | 5.70E-03 | 3.30E-03 | *NOTCH4(i)* |
| rs3806156 | 32481676 | 7.00E-04 | 4.40E-04 | 3.10E-03 | *BTNL2(i)* |
| rs3806157 | 32481779 | 9.00E-04 | 5.00E-04 | 3.60E-02 | *BTNL2(i)* |
| ***HLA* *DR*** | 32654524-32686031 | - | - | - | ***HLA DR*** |
| ***HLA* *DQB*** | 32735222-32754296 | - | - | - | ***HLA DQB*** |
| rs2071469 | 32892761 | 8.00E-05 | 4.40E-03 | 5.30E-02 | *HLA-DOB(5’) TAP2(d)* |
| rs210132 | 33644648 | 1.61E-03 | 4.40E-04 | 6.00E-02 | *BAK1(d)* |
| rs1570760 | 33730911 | 2.10E-03 | 2.00E-03 | 2.00E-02 | *ITPR3(i)* |
| rs1536036 | 33739992 | 2.60E-04 | 1.60E-04 | 1.30E-02 | *ITPR3(i)* |
| rs3904668 | 34571988 | 2.30E-03 | 2.80E-03 | 3.00E-02 | *PACSIN(i)* |

- = not applicable being the tag/conditioning allele.

Notation in brackets near gene names explain the location of the SNP in the gene context. (u): upstream; (d): downstream; (i): intronic; (5’): 5’untranslated
